# The Bias Associated with Amplicon Sequencing Does Not Affect the Quantitative Assessment of Bacterial Community Dynamics

**DOI:** 10.1371/journal.pone.0099722

**Published:** 2014-06-12

**Authors:** Federico M. Ibarbalz, María Victoria Pérez, Eva L. M. Figuerola, Leonardo Erijman

**Affiliations:** 1 Instituto de Investigaciones en Ingeniería Genética y Biología Molecular (INGEBI-CONICET), Buenos Aires, Argentina; 2 Agua y Saneamientos Argentinos S.A. (AySA), Buenos Aires, Argentina; 3 Departamento de Fisiología, Biología Molecular y Celular, Facultad de Ciencias Exactas y Naturales, Universidad de Buenos Aires, Buenos Aires, Argentina; Rockefeller University, United States of America

## Abstract

The performance of two sets of primers targeting variable regions of the 16S rRNA gene V1–V3 and V4 was compared in their ability to describe changes of bacterial diversity and temporal turnover in full-scale activated sludge. Duplicate sets of high-throughput amplicon sequencing data of the two 16S rRNA regions shared a collection of core taxa that were observed across a series of twelve monthly samples, although the relative abundance of each taxon was substantially different between regions. A case in point was the changes in the relative abundance of filamentous bacteria *Thiothrix*, which caused a large effect on diversity indices, but only in the V1–V3 data set. Yet the relative abundance of *Thiothrix* in the amplicon sequencing data from both regions correlated with the estimation of its abundance determined using fluorescence in situ hybridization. In nonmetric multidimensional analysis samples were distributed along the first ordination axis according to the sequenced region rather than according to sample identities. The dynamics of microbial communities indicated that V1–V3 and the V4 regions of the 16S rRNA gene yielded comparable patterns of: 1) the changes occurring within the communities along fixed time intervals, 2) the slow turnover of activated sludge communities and 3) the rate of species replacement calculated from the taxa–time relationships. The temperature was the only operational variable that showed significant correlation with the composition of bacterial communities over time for the sets of data obtained with both pairs of primers. In conclusion, we show that despite the bias introduced by amplicon sequencing, the variable regions V1–V3 and V4 can be confidently used for the quantitative assessment of bacterial community dynamics, and provide a proper qualitative account of general taxa in the community, especially when the data are obtained over a convenient time window rather than at a single time point.

## Introduction

The knowledge of the extent of bacterial diversity has expanded drastically since the introduction of culture-independent approaches based on molecular phylogenies of the small-subunit ribosomal RNA gene (16S rRNA gene). Amplicon-based bacterial community studies, in which a particular set of so-called universal PCR primers targets conserved regions flanking one or more of the nine variable regions (V1–V9) present in the 16S rRNA gene, have been extensively used to characterize the microbial community structures of several natural and engineered ecosystems. A problem with surveys of diversity performed using high-throughput amplicon sequencing is that the results are skewed by the bias associated with multi-template PCR reactions, no matter the sequencing depth [1]. Considerable attention has long been dedicated to investigate and minimize the many pitfalls of PCR-based estimates of microbial diversity [2], [3]. Sources of bias in the determination of “true” diversity include insufficient coverage of primers [4], [5], primer-template mismatches [6], [7], unequal amplification [8], [9], [10], and differential efficiency of annealing [6].

The choice of primers remains ultimately the most challenging issue for amplicon analysis [11], [12], [13], and there is still no consensus for the use of a particular 16S rRNA region [5], [12], [14], [15], [16]. Recent studies have provided valuable insight into the bias introduced by primer selection for the estimation of diversity using next generation sequencing in several ecosystems, such as soil [17], subgingival plaques [18], termite hindgut [19], human gut [20], [21] and activated sludge (AS) [22], [23]. These studies were primarily designed to determine which primer set could offer the most accurate taxonomic assignment of each microbiome. However, the purpose of 16S rRNA gene high-throughput surveys extends beyond the taxonomic profiling of microbial communities. It is also important to understand how microbial communities are structured in space [24] and time [25].

In this study we focused on the activated sludge bacterial populations dynamics. Activated sludge, which is used worldwide for wastewater treatment, comprises a diverse self-assembled and self-sustained microbial community. It has been suggested that patterns of bacterial community dynamics are likely regulated in part by operational parameters [26]. We asked whether different primer sets would be equally suitable for the characterization of the bacterial dynamics of a full-scale municipal wastewater treatment plant (WWTP).

The primary aims of this work were two-fold: First, to evaluate two of the most frequently used primers sets in high-throughput bacterial surveys for the estimation of the diversity metrics and taxonomic assignment of bacterial communities in temporally spaced samples of activated sludge, and secondly to assess how the bias introduced by the use of primer sets targeting different 16S rRNA gene regions affected the characterization of the dynamics of bacterial communities.

We hypothesized that even though the amplification of each region of the 16S rRNA gene produces a biased estimate of the community composition, they may still provide an accurate account of the population dynamics. To test this hypothesis, we performed high-throughput amplicon sequencing of the V1–V3 and the V4 variable regions of the 16S rRNA gene on activated sludge samples from a time series spanning one year of full-scale operation, and compared the performance of both pairs of data sets in describing the changes in the diversity and the temporal turnover of taxa.

## Materials and Methods

### Ethics statement

AySA is a state-owned company that provides drinking water and sewage services to the city of Buenos Aires and several districts of the Province of Buenos Aires. The permission for the collection and analysis of activated sludge samples of the AySA WWTP is included in the cooperation agreement between AySA and the Argentine National Research Council (CONICET), Res. 3816/11.

### Wastewater treatment plant description

The full-scale municipal WWTP is located in San Fernando, a suburban area of the city of Buenos Aires. The plant provides preliminary, primary and secondary treatment to remove organic matter and suspended solids for a population of 270,000 residents. Wastewater from primary clarifiers (0.9 m^3^/s) flows into three aerobic tanks that operate in parallel, each with a volume of 3350 m^3^. The mixed liquor from the aeration basins is combined before entering four secondary clarifiers. Main features of the treatment facility and influent wastewater are given on table S1 in File S1. Operational parameters of the WWTP were obtained from the staff members.

Samples from one of the aeration basins were taken on a monthly basis over a period of one year, starting on May 2012. Sludge samples were transported within 2 h to the laboratory in plastic flasks at room temperature with a large air chamber in order to avoid anaerobic conditions. Aliquots of 300 µl were fixed with 4 % paraformaldehyde for fluorescence in situ hybridyzation (FISH) analysis, and 2 ml were stored at −20°C.

### DNA isolation

DNA was extracted by a procedure involving physical disruption of cells and purified by the CTAB method. Shortly, pellets from 1.5 ml sludge were resuspended in 500 µl of TENP buffer (pH 7.6) and transferred to 2-ml screw-capped tubes with 200 µl of 0.5 mm zirconia/silica beads (BioSpec Products, Inc.). 50 µl of 10 % sodium dodecyl sulfate (SDS), 1 µl of RNAse A (100 mg/ml) and 3 µl proteinase K (20 mg/ml) were added, and incubated at 37°C for 2 h. After incubation, cells were physically disrupted and DNA extraction protocol was continued as described [27].

### PCR and sequencing

Variable V1–V3 and V4 regions of the 16S rRNA gene were amplified in duplicate from total DNA of all samples in the time series with universal primers F8 (5′-AGAGTTTGATCCTGGCTCAG-3′) and R534 (5′-WTTACCGCGGCTGCTGG-3′), and F563 (5′-AYTGGGYDTAAAGNG-3′) and R907 (5′-CCGTCAATTCMTTTRAGT-3′). The resulting 48 PCR amplicons (12 time points × 2 16S rRNA gene regions × 2 technical replicates) were tagged prior to sequencing using a 10 base pair multiplex identifier (MID), and sequenced in a Roche 454 GS FLX instrument at the Microbiome Core Facility (North Carolina, US). A total of 281,102 raw reads with an average length of 451 bp was generated for V1–V3 region, and 255,145 raw reads with an average length of 364 bp for V4 region. After the filtering procedure, these numbers were reduced to 161,822 and 140,753 reads, respectively (Table S2 in File S1).

Raw reads were submitted to the NCBI Sequence Read Archive under accession number SRP035875.

### Data analysis

Mothur v.1.31.2 was used to denoise, trim, filter and align sequences, find chimeras, assign sequences to operational taxonomic units (at 97 % similarity), and describe α-diversity [28], following the standard operating procedure suggested by the program's author. After extracting the flow files from the raw Standard Flowgram Format (sff) files, we applied the AmpliconNoise algorithm [29] to denoise the data. Sequences were quality-filtered (minimum length 200 bp, with no ambiguous bases and no more than 1 and 2 mismatches to the barcode and primer, respectively, and homopolymers of 8 bp as a maximum), separated by tag and trimmed. Sequences were aligned to the SILVA-database reference alignment v 102. Sequences not aligning in the targeted region were removed using the ‘screen.seqs’ command in mothur. Chimeras identified with the ‘uchime’ algorithm were also removed. The remaining sequences were alternatively (a) classified against 16S rRNA RDP database (training set 9) [30] with a bootstrap cutoff of 80 %, or (b) clustered into OTUs (97 % similarity) by average neighbor linkage, or (c) used to build a *de novo* tree using the program ‘clearcut’ implemented in mothur. In order to avoid the bias caused by differences in sequencing depth in the estimation of alpha- and beta-diversity, a subset of 2125 sequences were randomly subsampled from each replicate using mothur’s ‘sub.sample’ function.

OTUs defined at 97 % similarity had to be present in all twelve time points to be considered part of the core community. In order to allow comparison between the core populations detected by the two variable 16S rRNA regions, OTUs were classified against the RDP database. Because not all OTUs could be classified at the taxonomic level of genus, OTUs belonging to V1–V3 and V4 region with significant correlations between them (p < 0.01) were analyzed jointly through *blastn*, and queries with 100 % identity were added to the shared core. Ultimately, the shared core was established at the taxonomic level of order.

On the basis of the defined OTUs, we built rarefaction curves with a sampling iteration of 1000, and calculated Shannon index and observed OTUs for all samples. Diversity estimations from the different 16S rRNA regions were compared using linear mixed models with time as random factor, and a significance level of p < 0.05. Additionally, we obtained the Bray-Curtis dissimilarity matrix. Finally, we prepared *de novo* trees for each 16S rRNA region, which were analyzed separately to calculate weighted and unweighted UniFrac distances between samples [31].

Temporal shifts in bacterial community structure were evaluated from the distances or dissimilarities between successive dates along the temporal scale, a method known as a ‘moving-window’ analysis [32].

Species-time relationship (S  =  cT^w^) was estimated by adding either new genera or new OTUs at each time point to the respective initial count, and this was plotted against time on a log-log space. The bacterial replacement rate *w* was obtained from the slope of the linear regressions. OTUs represented by a single sequence in the whole data set were removed. Temporal similarity decay was analyzed as in [25]. Distances or dissimilarities were converted to similarity and then log-transformed. We performed independent linear regressions on the duplicate sets of data, obtaining two slope values for each region and each of three measures: Bray-Curtis dissimilarity, weighted and unweighted UniFrac distances. Slopes were compared using unpaired two-tailed Student's t test, with a significance level of p < 0.05.

Nonmetric Multidimensional Scaling (NMDS), Canonical Analysis of Principal Coordinates (CAP), Procrustes analysis and Mantel test were performed with the ‘vegan’ package version 2.0–10 (Department of Statistics, Iowa State University, Ames, IA, USA) in R 3.0.2, using default parameters.

CAP is a constrained ordination method that enables the use of non-Euclidean distances [33]. It was applied to assess the relationship between process variables and bacterial community dynamics, using V1–V3 and V4 average weighted UniFrac distances matrices as input. An ANOVA-like permutation test was used to assess the significance of constraints. The following explanatory variables were considered: mixed liquor suspended solids (MLSS), sludge volumetric index (SVI) and temperature (T) of the aeration basin, and biological oxygen demand (BOD) of the primary clarifier effluent (influent of the aeration basin).

### Fluorescence in situ hybridization

DAPI (4,6-diamidino-2-phenylindole) staining and in situ hybridization were performed in gelatin-treated glass slides, using a specific Cy3-labeled probe for *Thiothrix*, G123T (5′-CCTTCCGATCTCTATGCA-3′), in the presence of the competitor oligonucleotide 5′-CCTTCCGATCTCTACGCA-3′, in 40 % formamide [34]. After coating slides, a volume of paraformaldehyde-fixed sample (5 µl) was applied three times on the microscope slide and covered with agarose [35]. Negative controls were performed using the probe NONEUB (5′-ACTCCTACGGGAGGCAGC-3′) to monitor nonspecific binding [36].

### Image acquisition and analysis

Slides were examined with a Confocal Laser Scanning Microscope (CLSM) Olympus Fluoview FV1000, using an objective lens with a magnification of 600X (UPLSAPO 60X W NA:1.20), coupled to a CCD camera. We acquired digital images (512×512 Pixel) of 30 fields of view (FOV) at randomly chosen positions. For each randomly chosen FOV, we captured one image of the population-specific FISH probe signal (Cy3) and one image of the DAPI signal. The ratio between Cy3 and DAPI signals was determined on every FOV, and the average value was considered as an estimate of the volume that the detected taxon occupied in the sludge sample (biovolume). Image analysis and biovolume calculation were carried out with Daime 2.0 [35], downloaded from http://www.microbial-ecology.net/daime/.

## Results

### Taxonomic composition analysis

**Figure 1:**
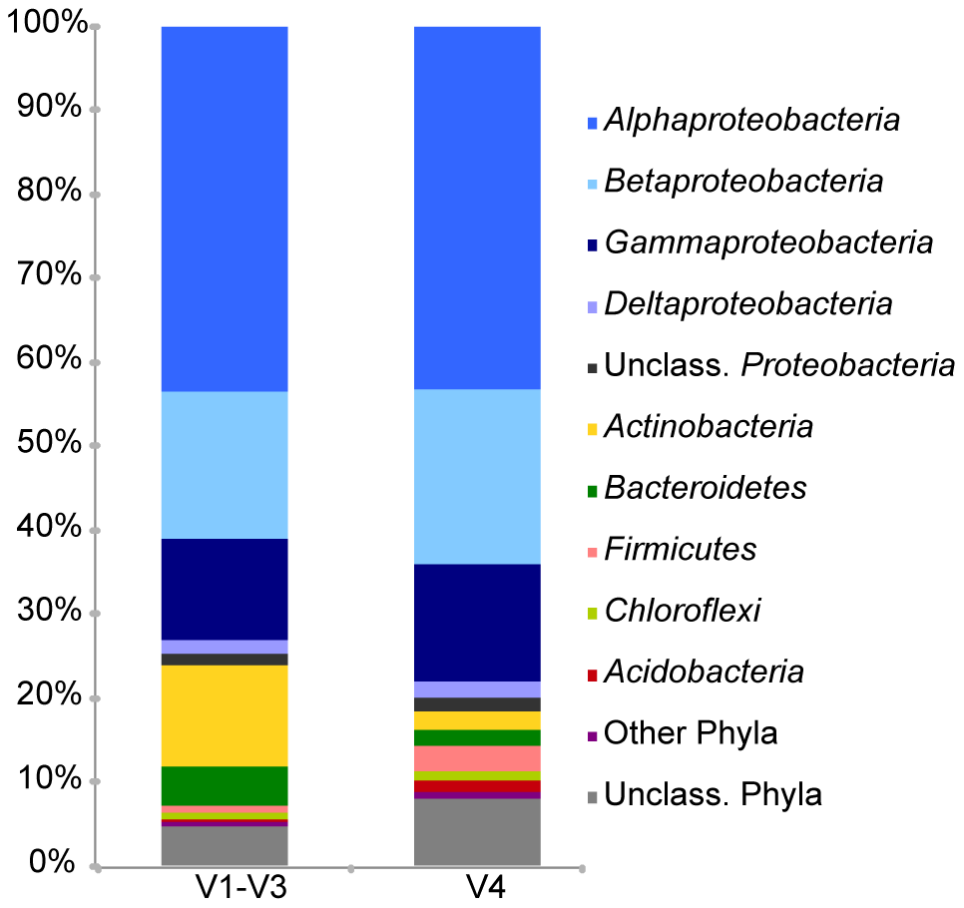
Distribution of bacterial phyla and classes of *Proteobacteria* according to the 16S rRNA gene region. Data of each 16S rRNA region correspond to the average of 12 duplicate monthly samples. Sequences were classified against RDP database at a confidence threshold of 80%. Phyla with average percentage of abundances lower than 1% were included in “other Phyla” (*Spirochaetes*, *Armatimonadetes*, *Epsilonproteobacteria*, SR1, *Deinococcus-Thermus, Synergistetes*, *Fusobacteria*, *Verrucomicrobia*, *Gemmatimonadetes*, TM7 and *Planctomycetes*).

**Figure 2:**
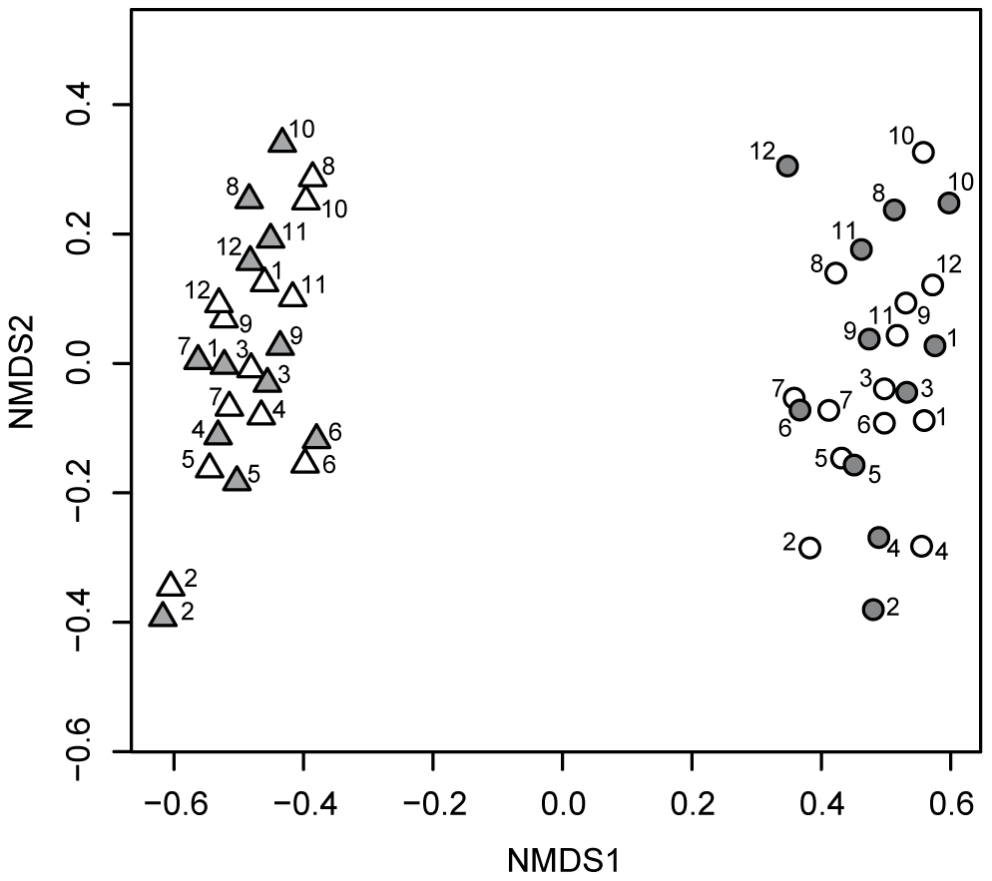
Nonmetric multidimensional scaling based on classified sequences at the genus level. Symbols represent each of the time points corresponding to V1–V3 region (▵) and to V4 region (○). Technical replicates are represented with the same symbols, but different filling (white and gray). The adjoining numbers identify the samples. Stress  =  0.13.

Temporally spaced AS samples, collected from a full-scale municipal wastewater treatment plant, were subjected to amplicon sequencing using different pairs of primers targeting, respectively, the variable regions V1–V3 and V4 of the 16S rRNA gene, each in duplicate. Bacterial community profiles showed that both regions detected *Proteobacteria* as the dominant phylum, but differed in the relative abundance of *Actinobacteria*, *Bacteroidetes, Firmicutes and Acidobacteria* (Fig. 1). The former two were more abundant in the V1–V3 region and the latter two were more represented in the V4 region. Bacteria belonging to *Chloroflexi* were found as minor members of the community in the two sets of data. A minor phylum, the candidate phylum SR1, was only detected in the V1–V3 reads, whereas sequences affiliated with phylum *Deinococcus*-*Thermus* were found exclusively in amplicons from the V4 region (Table S3 in File S1). Nonmetric multidimensional analysis showed a clear distribution of samples along the first ordination axis according to the sequenced region rather than according to sample identities (Stress  =  0.13, Fig. 2). The majority of the most abundant RDP-assigned genera were detected by both sets of primers, albeit they make up different proportions in each data set, resulting in biased community structures (Fig. S1 in File S1)

### Alpha-diversity

Rarefactions curves for 2125 randomly subsampled pyrosequencing reads did not reach saturation for V1–V3 nor for V4 amplicons (Fig. S2 in File S1). Richness derived from observed OTUs for the V1–V3 region was significantly higher than for the V4 region (355±55 and 284±36, respectively; p < 0.0001; Fig. S3A in File S1). Shannon index of V1–V3 region was also higher, although differences were not significant (4.31±0.48 and 4.23±0.25; p  =  0.27; Fig. S3B in File S1).

**Figure 3:**
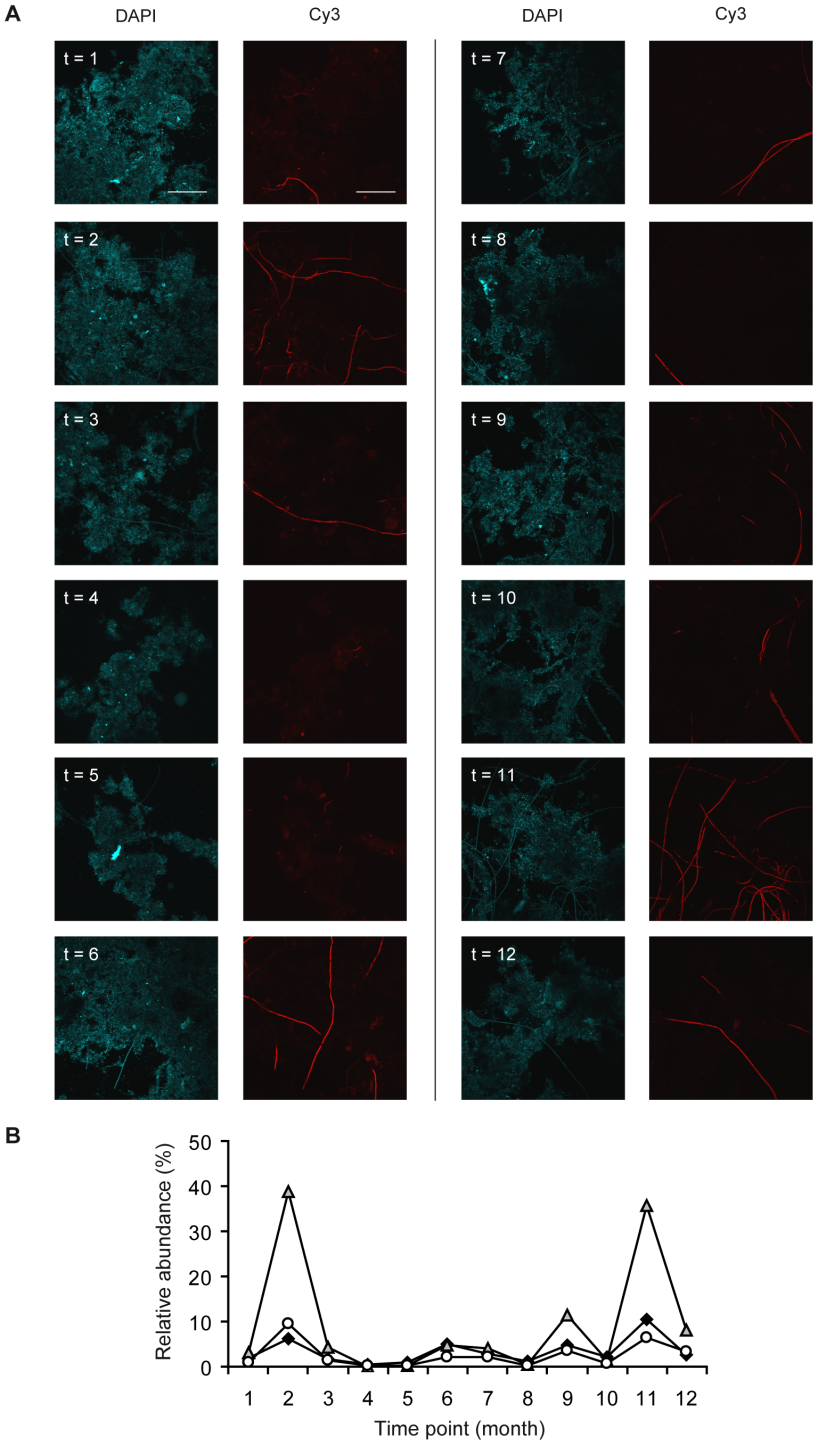
Quantification of *Thiothrix* sp. in activated sludge. (A) Representative images of fluorescence in situ hybridization of activated sludge at times 1 to 12. Two images of the same microscopic field are shown for each time point. Right panels: cells binding to *Thiothrix-specific* Cy3-labeled G123T probe. Left panels: corresponding views of DAPI stained cells. Photomicrographs were acquired in a CSLM at a magnification of 600X. Scale bar  =  50 µm, applies to all panels. (B) Biovolume fraction of *Thiothrix* relative to total bacteria determined by FISH (⧫). Relative abundances of *Thiothrix* sp determined by amplicon sequencing using the V1–V3 region (▵) and the V4 region (○).

There were two samples (2nd and 11th), in which Shannon index of region V1–V3 was lower than that of region V4. Those samples were characterized by high abundance of *Thiothrix*-related sequences, especially in the V1–V3 dataset. As a matter of fact, both primers pairs were able to amplify 16S rRNA fragments belonging to the genus *Thiothrix*, but exhibited a striking difference in the proportion of reads. Lack of amplification of *Thiothrix* species due to primer mismatch in the V4 region can be discarded on the basis that V4-forward F563 and V4-reverse R907 primers match 98.5 % and 97 %, respectively, of the sequences > 1200 bp of *Thiothrix* in the RDP database. Since the relative abundance of *Thiothrix* introduced a large weight on the diversity metrics, we decided to compare the relative abundance of *Thiothrix* in the pyrosequencing datasets of both regions with a quantitative estimation obtained using fluorescence in situ hybridization (Fig. 3A). The biovolume fraction of *Thiothrix* in the 12 samples of the time series ranged between 0.4–10.6 %, in closer agreement with the values obtained by sequencing the amplicons of the V4 region (Fig. 3B). Yet the biovolumes occupied by *Thiothrix* determined by FISH were not only correlated with the relative abundance of *Thiothrix* estimated from the sequencing of the V4 region (r  =  0.81), but also with the one corresponding to V1–V3 region (r  =  0.87).

### Temporal core microbiome

**Figure 4:**
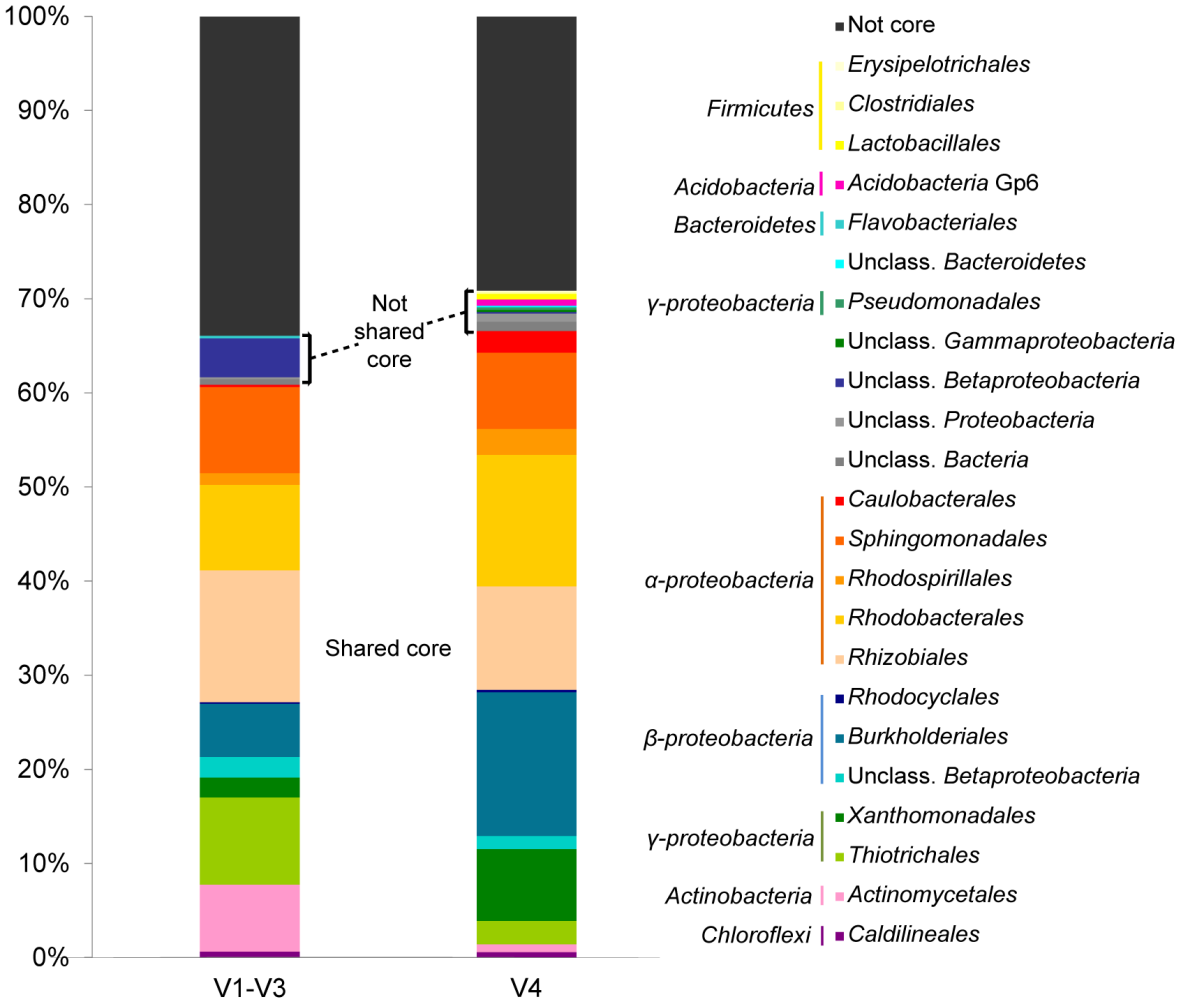
Composition of the temporal core of activated sludge samples. Distribution of bacterial orders within the core microbiome. Data of each 16S rRNA region correspond to the average of 12 duplicate monthly samples.

We noted that both sets of data contained a collection of OTUs that were observed across all sampling dates, suggesting the existence a temporal “core” microbiome within the WWTP. We compared the taxonomic affiliation of the OTUs in the core community detected by each of the sequenced regions, classified at the taxonomic level of order (Fig. 4). Almost two thirds of the OTUs total abundance corresponded to core OTUs shared by both regions. However, each of those OTUs was represented in the core community with different average relative abundance. Fig. S4A in File S1 shows the changes over time of members of the core using V1–V3 and V4 regions. A moving-window analysis followed by a Pearson correlation indicated that the fluctuations of taxa within the core were not significantly correlated (r =  0.57, p = 0.07; Fig. S4B in File S1).

### Beta-diversity

The matrices describing the dissimilarity (distance) between all pair of bacterial communities in each time series were highly correlated for the V1–V3 and V4 regions. That was true using a taxonomic dissimilarity index (Bray Curtis, Mantel test, r  =  0.85, p < 0.001) and phylogenetic metrics (weighted UniFrac, r  =  0.82, p < 0.001, and unweighted UniFrac, r  =  0.82, p < 0.001). Coincidently, Procrustes analysis indicated that NMDS ordinations based on V1–V3 and V4 regions were highly correlated (corr.  =  0.90, p < 0.001; Fig. S5 in File S1).

**Figure 5:**
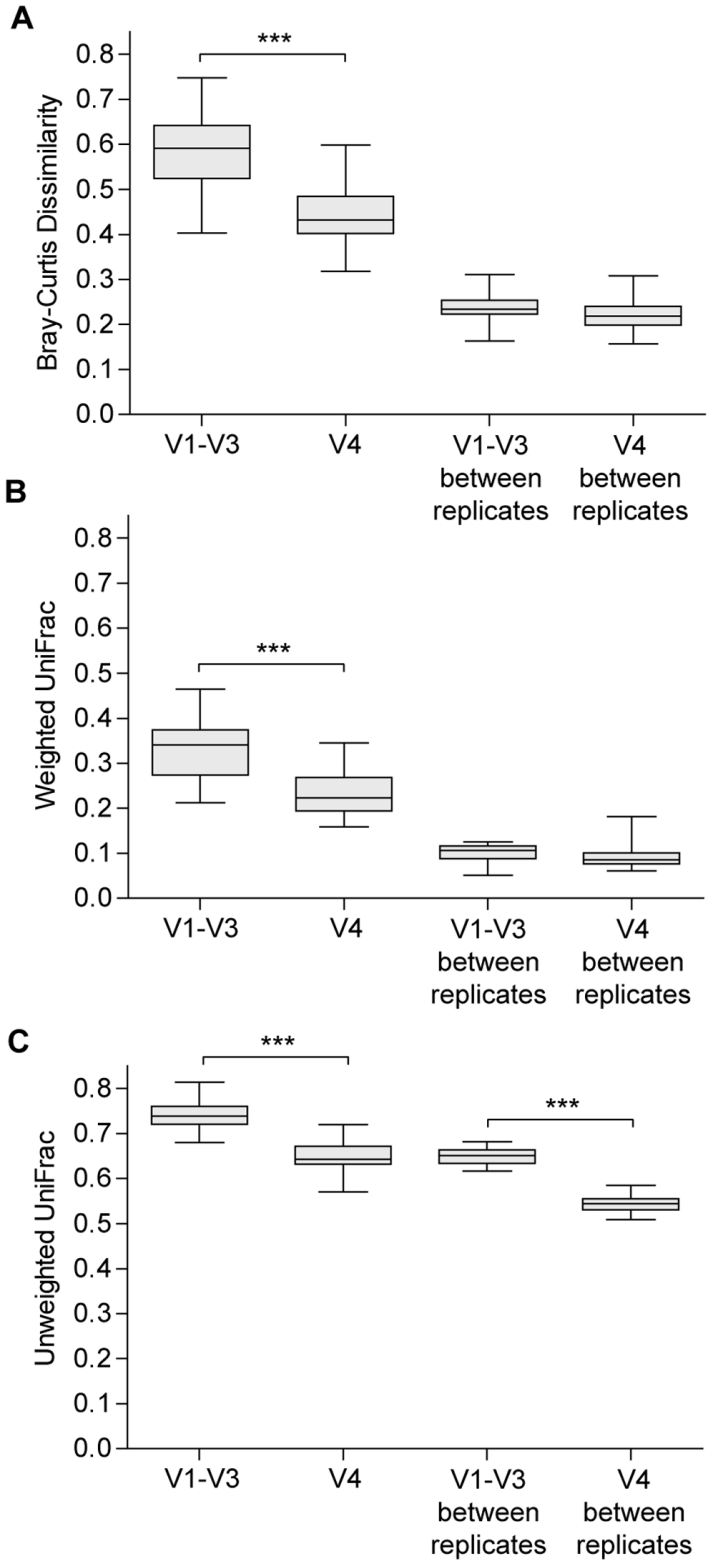
Pairwise comparisons between all samples in each dataset. Boxplot of median, range and interquartile range of (A) Bray-Curtis dissimilarity, (B) weighted UniFrac distance and (C) unweighted UniFrac distance.

Average distances (and dissimilarities) were significantly lower for V4 region compared to those of V1–V3 region (p < 0.001). Because communities obtained using V1–V3 amplicons had higher richness, it was necessary to rule out the possibility that the differences in beta diversity were due to random sampling variability. Fig. 5 shows that the average weighted UniFrac distances and Bray-Curtis dissimilarities of all pair of replicates were approximately the same for both 16S regions. Only the unweighted UniFrac metric, which is more influenced by the larger number of rare or unique species, was significantly higher for the V1–V3 region (p < 0.001).

### Bacterial community dynamics

We explored the influence of primer choice on the characterization of bacterial community dynamics. For that purpose, we used the matrices of Bray-Curtis dissimilarities (with OTUs defined at 97% similarity) or UniFrac distances of all pair of samples within V1–V3 or V4 datasets. Three types of analyses were performed: moving-window, similarity decay and species-time relationship.

### Moving-window analysis

**Figure 6:**
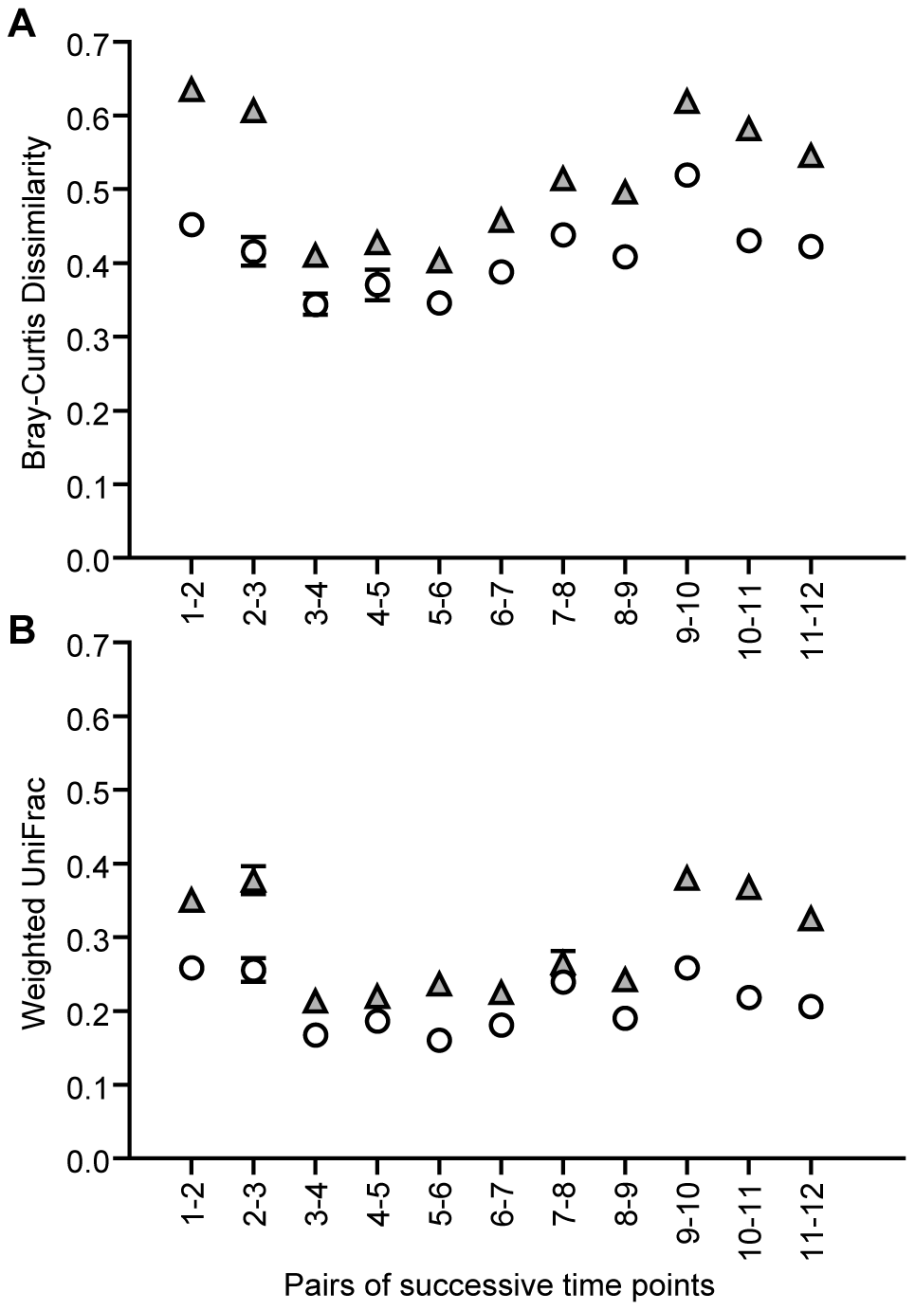
Moving-window analysis. Symbols indicate the mean of (A) Bray-Curtis dissimilarities or (B) weighted UniFrac distances between consecutive sampling points within the V1–V3 (▵) and the V4 (○) datasets. Error bars represent SEM.

The dynamics of microbial communities in the activated sludge community was initially monitored by measuring month-to-month variations in bacterial community dissimilarity (Fig. 6). Even though V1–V3 exhibited higher values of taxonomic and phylogenetic metrics, both 16S rRNA gene regions yielded comparable patterns of the changes occurring within the communities along the fixed time interval. This was valid for both Bray-Curtis dissimilarity and weighted UniFrac distance (Pearson coefficients, r  =  0.86, r  =  0.84, respectively).

### Similarity decay

**Figure 7:**
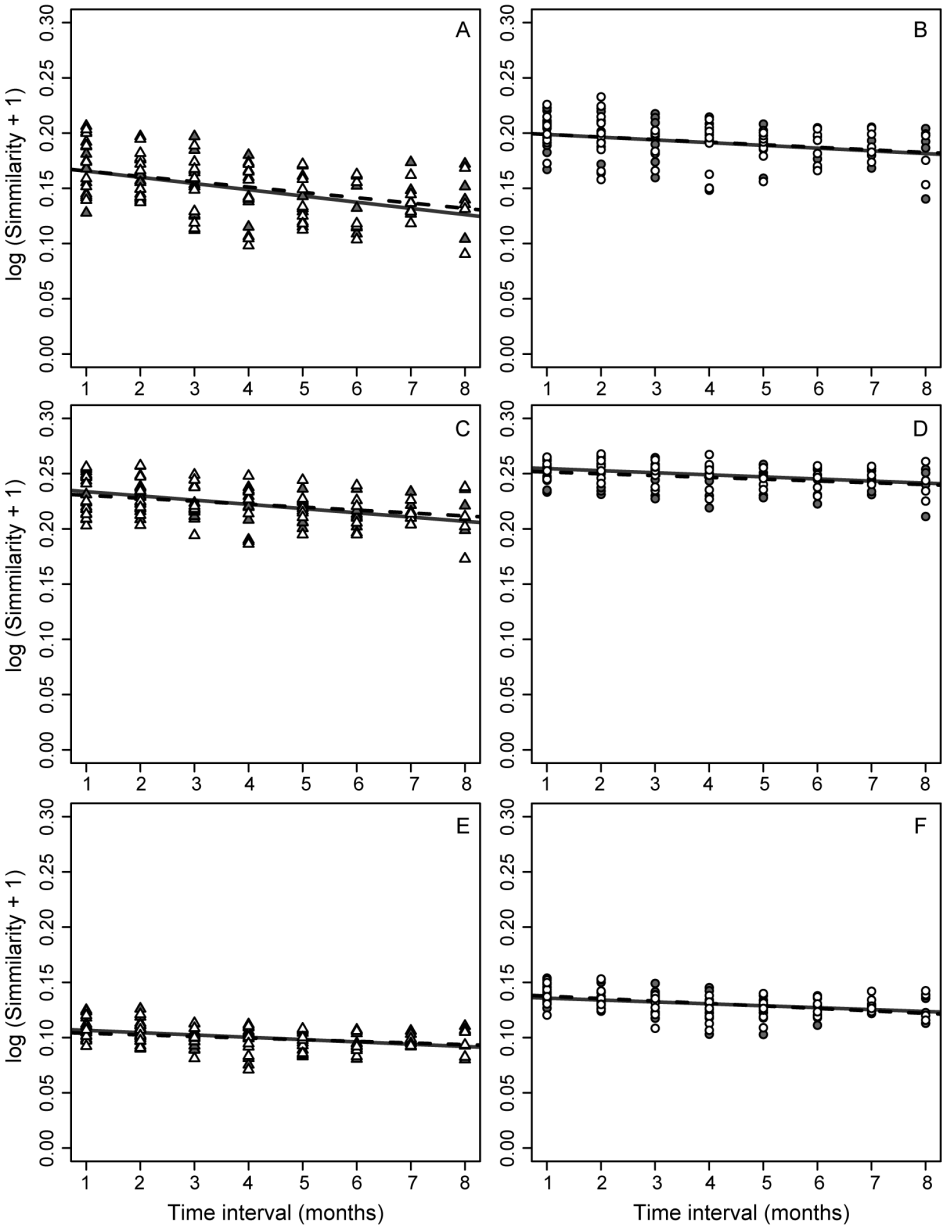
Impact of primer choice on the similarity decay. (A, B) Bray-Curtis dissimilarities, (C, D) weighted UniFrac, and (E, F) unweighted UniFrac distances, were converted to similarities and fitted to a log-linear model. Symbols represent each of the pairwise comparisons according to V1–V3 region (▵) and to V4 region (○). Technical replicates are represented with the same symbols, but different filling (white and gray). Linear regressions were calculated independently for each replicate and plotted with continuous and dashed lines. Slopes derived from V1–V3 and V4 data sets were not significantly different.

To evaluate how the similarity between communities changed with increasing time, pairwise UniFrac distances and Bray-Curtis dissimilarities were converted to similarity values and plotted on a log scale against all time intervals between sampling. Linear regressions had slightly negative slopes (p < 0.05 in all cases). The differences in slopes between both regions were not significant for any of the similarity metrics, indicating that the two 16S rRNA regions can depict equally well the relatively slow turnover of AS communities (Fig. 7).

### Species-time relationship

**Figure 8:**
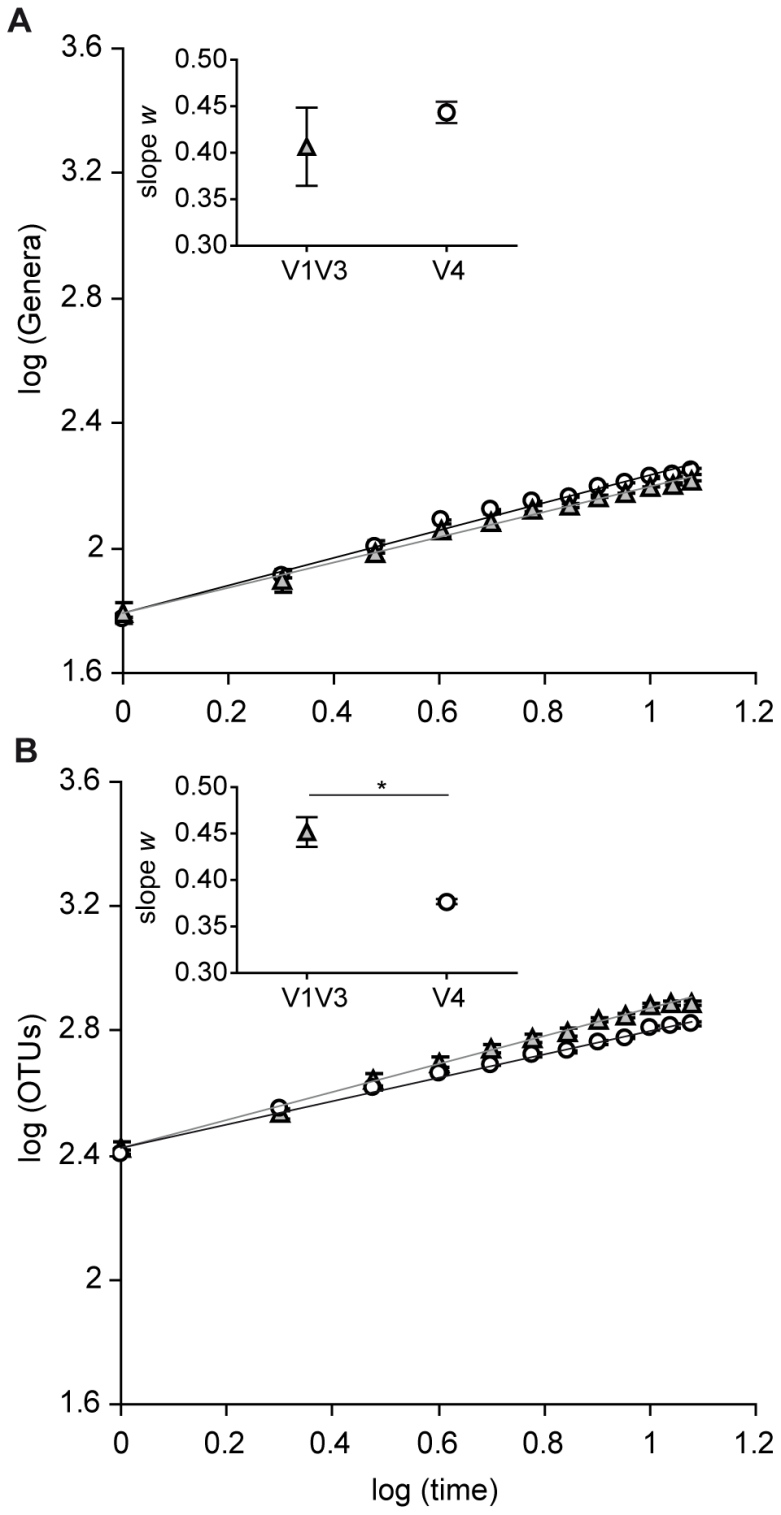
Impact of primer choice on bacterial turnover. The rate of species replacement (*w*) was calculated on the basis of (A) the classified sequences and (B) OTUs with a cutoff of 97% similarity. Symbols represent the average values for each time point according to V1–V3 region (▵) and to V4 region (○). Error bars in the log-log space represent SEM of log values.

Community dynamics was ultimately analyzed through the species-time relationship, which describes by the power law equation S  =  cT*^w^* how the number of species rises with increasing time of observation. The bacterial taxa–time relationships were displayed in a log–log space plot (Fig. 8). The average of exponent *w* of the V1–V3 region calculated using classified sequences (0.41±0.06) was very close to the average exponent calculated from that of the V4 region (0.44±0.02, p  =  0.49). The OTU-based analysis yielded for the V1–V3 region a slightly higher exponent *w* (0.45±0.02) than the one calculated from the data of the V4 region (0.377±0.004, p  =  0.04).

### Community structure and WWTP operation

**Figure 9:**
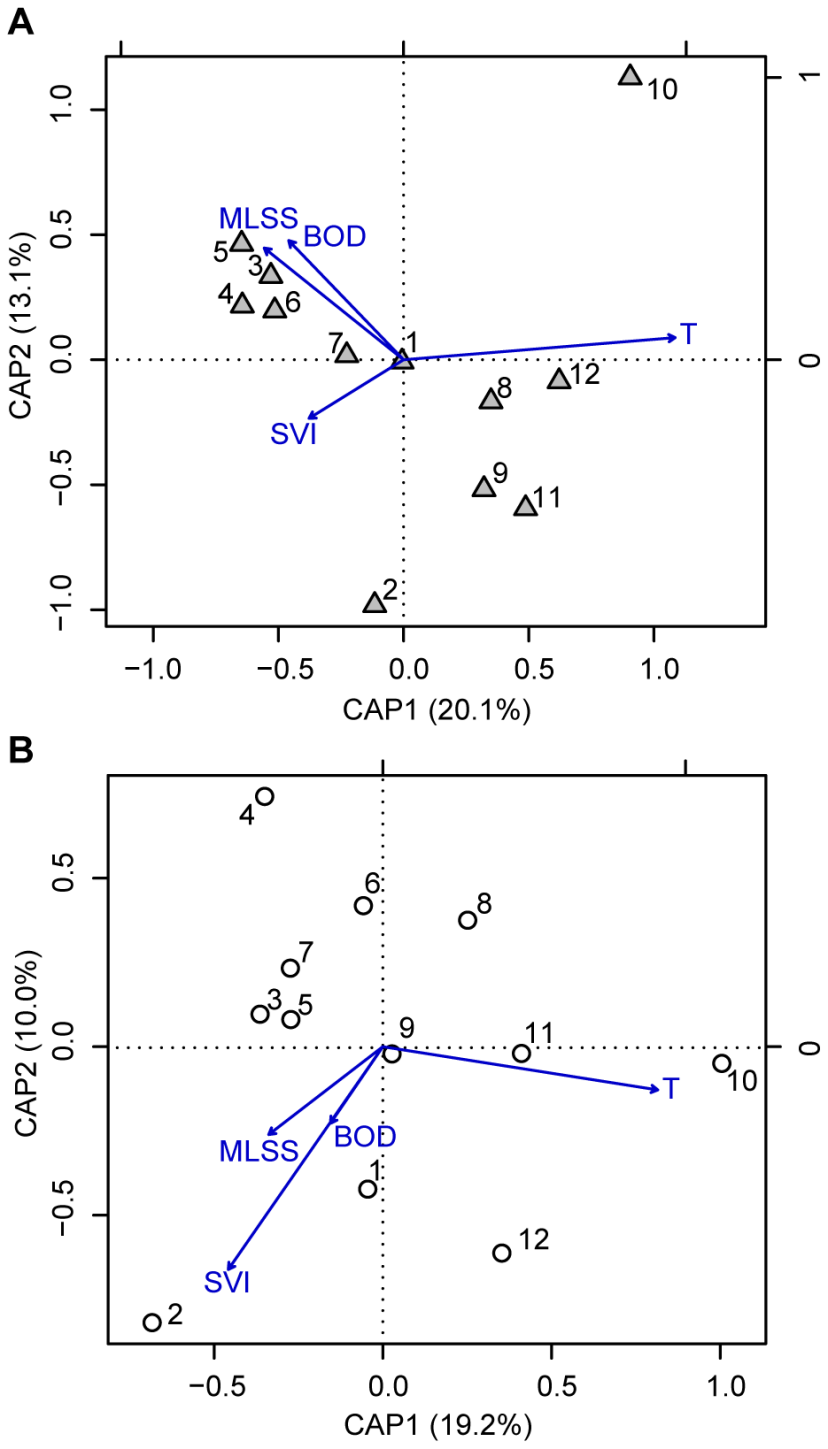
Constrained Analysis of Principal Coordinates (CAP). CAP was performed using the average weighted UniFrac distances and four measured operational parameters: temperature, mixed liquor suspended solids (MLSS), sludge volume index (SVI) and influent biochemical oxygen demand (BOD). (A) V1–V3 region; (B) V4 region.

A Canonical Analysis of Principal Coordinates (CAP) was applied to examine the influence of targeting different regions on a constrained ordination (Fig. 9). There is no clear pattern in terms of the distribution of samples along the principal coordinate axes in the constrained analyses. The temperature was the only operational variable that showed significant correlation with bacterial communities over time (p  =  0.01 for both regions).

## Discussion

Our results show that despite giving a biased estimation of bacterial diversity, different 16S rRNA gene primer sets used for high-throughput amplicon sequencing provide a similar quantitative measure of activated sludge bacterial population dynamics. This is important because in diverse and dynamic microbial ecosystems detecting and understanding temporal patterns may be more informative than knowing the identity of the individual populations.

It is well known that in PCR-based metagenomic analyses the selection of the 16S rRNA gene region that is amplified carries a potential source of bias for the estimation of diversity [1], [11], [17], [18], [19], [20], [21], [22], [23]. In most studies, comparison between samples is appropriately validated by the fact that all samples are subjected to the same biases. We have detected consistently higher richness estimations for V1–V3 data, likely based on the fact that V4 has a lower coverage due to the reduced conservation at flanking sites [37]. However, the matrices describing the dissimilarity between all pair of bacterial communities in each time series were highly correlated for both the V1–V3 and V4 regions

### Bacterial population dynamics in wastewater treatment

Still, the question remains as to whether the skewed representation of microbial diversity may lead to inaccurate reflections of the temporal scaling. Based on a growing body of literature on microbial community dynamics, it has been suggested that the variations through time exhibited by microbial communities are, as in plant and animal communities, likely influenced by a variety of abiotic and biotic factors [25], [38]. The temporal patterns could therefore be used to address ecological questions about mechanisms and processes [39], [40]. In the particular case of biological wastewater treatment, it has long been recognized that highly variable community structures can sustain stable process performance, most likely on the basis of the turnover of functionally equivalent species [26], [41], [42], [43], [44], [45], [46], [47]. Therefore, it is challenging to discriminate between shifts in community structure due to the natural, unperturbed dynamics from those caused by biotic and environmental variations [38], [40]. Important steps have been taken to advance our understanding of how the variability in community composition is shaped by key ecological factors in wastewater treatment, e.g. through the work performed in lab scale activated sludge bioreactors, which led to the discovery of a positive correlation between population dynamics and performance efficiency in full-scale bioreactors treating brewery wastewater [48], and also through the finding of the dependency of the turnover of bacterial species on the organic loading rate [49], and on the metacommunity size [50].

We show in this work that despite the differences in α-diversity metrics, amplicon abundance of both V1–V3 and V4 regions provide quantitatively equivalent measures of bacterial turnover. The exponent *w* of the power law STR, which characterizes the increase in the observed number of species with increasing time [39] was very similar for both regions, and falls within the range of previous surveys of other microbial, animal and plant communities [25]. Only the exponent calculated from the OTU-based analysis was slightly higher for the V1–V3 region, compared to the one calculated for the V4 region.

As pointed out by [51], several mechanisms can influence the slope of STR, including the fact that longer time periods allow the sampling of more individuals and incorporate increased environmental heterogeneity. Additionally species co-occurrence can be influenced by dispersal limitations or biotic interactions [51]. Therefore, although inferring causal explanation of STR curves will remain difficult, a suitable description of microbial population dynamics could help to disentangle the contribution of each of the individual factors.

The change between community profiles of consecutive time samples (moving-window analysis) appeared to follow a seasonal pattern, with higher variability occurring during the warm months. Directional changes in community composition in response to environmental conditions have not been universally observed in all microbial assemblages, but have been detected in several aquatic ecosystems [52], [53], [54]. Although longer time-series observations will be needed to confirm this trend, this result is consistent with the fact that temperature is the environmental variable that best explained the temporal changes in activated sludge bacterial community structure, a finding that has been reported before [26], [55], [56]. We note that although the correlation may be artificial because the samples are related by time, samples were not ordinated according a temporal gradient, but the maximum separation occurred along the first axis between winter samples, which had temperatures below 18°C and summer samples, with temperatures above 26°C.

Yet the very low rate of similarity decay across time intervals detected equivalently by both 16S rRNA regions indicated that the dynamic changes in the bacterial community did not continue to diverge with increasing time. This can be understood by the fact that approximately two third of the abundance in the community was constituted by a temporally stable “core”, whereas only rarer taxa exhibited higher variability. A similar pattern has already been observed in microbial communities involved in wastewater treatment, both aerobic [55] and anaerobic [48]. This is also consistent with the conclusions derived from a recent meta-analysis that included 76 bacterial and archaeal time series assessed via high-throughput sequencing of the 16S rRNA gene [25], which showed that microbial communities of brewery wastewater treatment [48] were less variable than most microbial assemblages. These observations have been interpreted in terms of the potentially important influence of deterministic processes that may lead to species sorting within wastewater treatment communities [25]. We agree with this hypothesis, based on the results presented in this work and on previous data from our and other laboratories, which suggest an important role for niche selection in the assembly of bacterial communities in biological wastewater treatment. In a survey of industrial and domestic activated sludge systems, we have shown that samples from geographically distributed WWTP clustered according to the type of treated wastewater rather than by geographic distance or operational conditions [57]. Additionally, a recent meta-analysis of 50 activated sludge samples from globally distributed full-scale WWTPs confirmed that bacterial assembly in AS was shaped by taxonomic relatedness and that bacterial co-occurrence at high taxonomic ranks was higher than expected by chance [58]. A different meta-analysis of 78 anaerobic digester samples originating from 28 different studies also found that digester microbiomes clustered by substrate type [59].

### Bias in the representation of the core community

Thus, the environmental filter leads to the recruitment of a core of bacterial populations, which are likely associated to the function of the community. Although the communities profiled by each of the 16S rRNA gene region were distinct from each other, both regions provided a qualitatively equivalent representation of the core constituents, whereas just minor OTUs were detected only by a single pair of primers. This is why it was still possible to find patterns of relatedness even in datasets that incorporated sequences covering different regions of the 16S rRNA [57], [59].

However, because of the different amplification efficiencies of particular species for each region [15], there were large differences in the proportional abundance of the core species, as well as in the detection of rare members of the community. As in previous studies of in vitro-simulated communities [15], we detected the presence of few sequences likely prone to PCR bias (*Thiothrix* for the V1–V3 region, *Thermomonas* and *Comamonas* for the V4 region), which can drastically skew the observed relative abundances of other members of the community. Additionally, clustering methods and taxonomic assignments can also affect the outcome of community structure analyses. Together, these differences are responsible for the separation of communities according to the amplicon region rather to the sample identity (Fig. 2, see also [17]).

Further pros and cons of using amplicon sequencing to quantify critical bacterial populations are illustrated by the case of *Thiothrix*. The activated sludge process is sensitive to the outgrowth of particular populations, which can be detrimental to the process performance. *Thiothrix* is a colorless sulfur-oxidizing filamentous bacteria that may cause bulking, one of the most common operational problem affecting activated sludge systems [60]. Data sets from both regions showed indeed large differences in the relative abundance of *Thiothrix*. In some of the samples the OTU assigned to *Thiothrix* had a disproportionately large effect on the Shannon index, but only in the V1–V3 data set. This case serves to illustrate how the preferential amplification of particular sequences may render the determination of diversity indices inaccurate.

Nevertheless, the relative abundance of *Thiothrix* in the amplicon sequencing data from both regions correlated with the estimation of *Thiothrix* abundance obtained using fluorescence in situ hybridization. Thus, even though amplicon sequencing does not provide a fully quantitative description of *Thiothrix* abundance, either region can still provide a suitable qualitative picture of their relative content. Future studies should aim to address whether this correlation holds beyond the organism studied here by analyzing a larger number of bacterial populations.

### Concluding remarks

In spite of the considerable effort already made to minimize the bias introduced in PCR-mediated determination of microbial diversity, there is broad consensus about the practical impossibility of completely achieving this goal. We show that despite this bias, amplicon sequencing can be confidently used for the quantitative assessment of bacterial community dynamics, and provides a proper qualitative account of general taxa in the community, especially when the data are obtained over a convenient time window rather than at a single time point. This is significant because it allows direct comparison between studies performed using different 16S rRNA regions. On the other hand, because of the distortion caused by the preferential amplification of particular sequences, it is recommended that quantitative characterization of critical bacterial populations rely on a truly quantitative method, such as FISH.

## Supporting Information

File S1 **Supporting Information file.** Table S1. Operational parameters of the wastewater treatment plant. Table S2. Numbers of reads obtained throughout the bioinformatic workflow. Table S3. Distribution of classified 16S rRNA sequences at the phylum level for 12 activated sludge samples (two technical replicates for each of the two variable regions). Figure S1. Heatmap of bacterial genera abundance in each time point, based on the analysis of the two 16S rRNA regions. The 25 most abundant genera are shown. Color scale refers to the square root of the abundance of each genus relative to all bacterial sequences. Figure S2. Estimates for bacterial richness are affected by the choice of primers. Rarefaction curves built for OTUs defined at 97% similarity. Each plot corresponds to a time point (month) for V1–V3 (▵) and V4 (○). Error bars show the 95% confidence interval derived from the 1000-time iterative sampling. Figure S3. Effect of the different primer sets on the estimation of alpha-diversity. (A) Observed richness and (B) Shannon index were calculated on the normalized samples as average between the two technical replicates. Gray bars represent the values obtained with primers F8-R534 (V1–V3) and white bars, with primers F563-R907 (V4). Error bars show SEM. Figure S4. Changes in the relative sequence abundance of bacterial core members. A) Time series of the relative abundances of core members for each 16S rRNA variable region data set. OTUs considered part of the core were detected in all twelve sampling points in at least one 16S rRNA region; all other OTUs were grouped as “not core”. Replicates within each 16S rRNA region were averaged for clarity of presentation. B) Moving-window analysis. Symbols indicate the mean of Bray-Curtis dissimilarities between consecutive sampling points within the V1–V3 (▵) and the V4 (○) core datasets. Error bars represent standard error. Figure S5. Comparison of beta diversity results based on V1–V3 and V4 16r RNA regions. Procrustes analysis of NMDS ordination plots based on V1–V3 and V4 datasets. Replicates within each 16S rRNA region were averaged for clarity of presentation. Blue lines connect paired samples on the target configuration indicated by circles (V1–V3 region), and the reference configuration (end of arrow, V4 region). Correlation *r* =  0.90, p < 0.001.
(PDF)
