# Supplementary material for: The Bias Associated with Amplicon Sequencing Does Not Affect the Quantitative Assessment of Bacterial Community Dynamics
Source: PLoS One. 2014 Jun 12;9(6):e99722. doi: 10.1371/journal.pone.0099722 (PMC4055690; doi:10.1371/journal.pone.0099722)
Supplement: File S1 — Supporting Information file. Table S1. Operational parameters of the wastewater treatment plant. Table S2. Numbers of reads obtained throughout the bioinformatic workflow. Table S3. Distribution of classified 16S rRNA sequences at the phylum level for 12 activated sludge samples (two technical replicates for each of the two variable regions). Figure S1. Heatmap of bacterial genera abundance in each time point, based on the analysis of the two 16S rRNA regions. The 25 most abundant genera are shown. Color scale refers to the square root of the abundance of each genus relative to all bacterial sequences. Figure S2. Estimates for bacterial richness are affected by the choice of primers. Rarefaction curves built for OTUs defined at 97% similarity. Each plot corresponds to a time point (month) for V1–V3 (▵) and V4 (○). Error bars show the 95% confidence interval derived from the 1000-time iterative sampling. Figure S3. Effect of the different primer sets on the estimation of alpha-diversity. (A) Observed richness and (B) Shannon index were calculated on the normalized samples as average between the two technical replicates. Gray bars represent the values obtained with primers F8-R534 (V1–V3) and white bars, with primers F563-R907 (V4). Error bars show SEM. Figure S4. Changes in the relative sequence abundance of bacterial core members. A) Time series of the relative abundances of core members for each 16S rRNA variable region data set. OTUs considered part of the core were detected in all twelve sampling points in at least one 16S rRNA region; all other OTUs were grouped as “not core”. Replicates within each 16S rRNA region were averaged for clarity of presentation. B) Moving-window analysis. Symbols indicate the mean of Bray-Curtis dissimilarities between consecutive sampling points within the V1–V3 (▵) and the V4 (○) core datasets. Error bars represent standard error. Figure S5. Comparison of beta diversity results based on V1–V3 and V4 16r RNA regions. Procrustes anal [file pone.0099722.s001.pdf]

Table S1. Operational parameters of the wastewater treatment plant.

	Range	Average	SD
Primary settling tank effluent BOD[a] (mg/l)	61-125	87	24
Primary settling tank effluent COD[b] (mg/l)	153-271	204	45
Mixed liquor temperature (°C)	17.1-26.6	20.8	3.3
MLSS [c] (mg/l)	933-2593	1892	487
Mixed liquor pH	7.10-7.67	7.37	0.2

Average solids retention time (SRT) = 7 days

[a] Biological oxygen demand
[b] Chemical oxygen demand
[c] Mixed liquor suspended solids

Table S2. Numbers of reads obtained throughout the bioinformatic workflow.

	**V1-V3**	**V4**	**All**
Raw sequences	281,102	255,145	536,247
Trim flow files	198,750	146,525	345,275
Trim denoised fasta files	198,612	146,489	345,101
Align and remove non V1-V3 or V4 sequences	193,713	145,101	338,814
Remove chimeras	161,829	140,770	302,599
Remove contaminants	161,822	140,753	302,575

Table S3. Distribution of classified 16S rRNA sequences at the phylum level for 12 activated sludge samples (two technical replicates for each of the two variable regions)

Sample*	*Acidobacteria*	*Actinobacteria*	*Armatimonadetes*	*Bacteroidetes*	*Chloroflexi*	*Deinococcus-Thermus*	*Firmicutes*	*Fusobacteria*	*Gemmatimonadetes*	*Nitrospira*	*Planctomycetes*	*Proteobacteria*	SR1	*Spirochaetes*	*Synergistetes*	TM7	*Verrucomicrobia*	Unclassified bacteria
V1V3_1A	15	385	0	72	27	0	14	0	0	0	29	1406	0	0	0	2	1	174
V1V3_1B	1	380	0	49	39	0	24	0	0	0	30	1448	0	0	0	2	0	152
V1V3_2A	1	413	0	8	35	0	10	0	0	0	6	1570	2	0	2	8	0	70
V1V3_2B	2	410	1	5	42	0	8	0	1	0	3	1592	4	0	0	3	0	54
V1V3_3A	5	275	0	83	15	0	21	0	2	0	8	1647	0	0	0	3	0	66
V1V3_3B	4	264	0	86	23	0	25	0	1	0	7	1624	0	0	1	3	0	87
V1V3_4A	3	497	0	75	4	0	47	1	0	0	5	1432	0	0	1	2	0	58
V1V3_4B	0	480	0	80	5	0	17	6	0	0	8	1482	0	0	1	1	0	45
V1V3_5A	0	300	0	73	3	0	13	0	0	0	1	1622	6	0	0	29	1	77
V1V3_5B	1	294	1	69	3	0	19	1	0	0	1	1659	0	0	1	22	0	54
V1V3_6A	1	82	0	49	2	0	17	0	0	0	0	1927	1	0	0	6	0	40
V1V3_6B	0	77	0	61	4	0	22	0	0	0	0	1882	2	0	1	13	0	63
V1V3_7A	2	221	0	34	4	0	21	1	0	0	0	1763	0	0	1	4	0	74
V1V3_7B	0	194	0	38	2	0	28	0	0	0	4	1782	0	0	0	2	0	75
V1V3_8A	4	215	0	130	25	0	21	0	1	0	11	1550	0	0	0	0	2	166
V1V3_8B	2	193	0	115	13	0	24	0	2	0	6	1605	0	0	0	1	1	163
V1V3_9A	0	203	0	92	2	0	12	0	0	0	1	1742	5	0	0	2	0	66
V1V3_9B	0	248	0	59	5	0	14	0	0	0	3	1725	3	0	0	0	0	68
V1V3_10A	12	188	1	300	57	0	13	0	5	1	8	1336	0	3	0	2	0	199
V1V3_10B	8	220	0	366	43	0	16	0	2	0	14	1251	0	2	0	2	1	200
V1V3_11A	0	149	1	47	29	0	18	0	2	0	5	1749	1	0	1	1	0	122
V1V3_11B	1	149	0	61	25	0	12	0	0	0	5	1790	0	0	1	1	0	80
V1V3_12A	1	194	0	234	2	0	9	0	3	0	4	1590	1	0	0	1	0	86
V1V3_12B	2	152	0	239	5	0	11	0	0	0	7	1631	0	0	0	5	0	73
V4_1A	95	63	0	14	35	1	53	0	0	0	16	1672	0	0	2	0	4	170
V4_1B	81	55	0	38	46	1	48	0	1	0	34	1621	0	1	0	0	3	196
V4_2A	12	77	0	47	49	2	95	0	0	0	18	1581	0	0	0	0	0	244
V4_2B	47	116	1	16	67	2	148	9	0	0	7	1503	0	0	0	0	0	209
V4_3A	31	38	1	29	17	0	70	2	2	0	4	1697	0	0	1	1	3	229
V4_3B	28	24	0	36	26	1	69	2	3	0	8	1734	0	0	4	0	1	189
V4_4A	16	33	0	26	15	5	129	6	0	0	5	1749	0	0	1	0	1	139
V4_4B	16	47	2	47	5	8	112	12	3	0	10	1645	0	0	1	1	3	213
V4_5A	14	46	0	41	11	0	72	5	0	0	2	1818	0	0	2	1	0	113
V4_5B	16	34	0	39	8	0	76	3	0	0	2	1854	0	0	1	2	0	90
V4_6A	41	30	0	10	5	0	76	4	1	0	1	1829	0	1	1	4	0	122
V4_6B	27	20	0	34	7	0	62	2	1	0	1	1834	0	0	1	1	4	131
V4_7A	5	35	0	19	6	0	74	1	1	0	1	1894	0	0	1	1	1	86
V4_7B	12	41	0	28	11	0	83	1	1	0	1	1846	0	0	0	3	1	97
V4_8A	20	39	0	85	15	0	28	0	6	0	11	1665	0	0	0	0	4	252
V4_8B	36	27	0	50	20	1	42	1	8	0	4	1666	0	0	0	0	4	266
V4_9A	9	77	0	53	4	3	30	0	7	0	7	1843	0	0	0	0	5	87
V4_9B	11	48	0	29	10	0	47	0	2	0	3	1906	0	0	1	0	0	68
V4_10A	70	32	0	57	60	0	45	0	16	0	8	1571	0	0	0	0	4	262
V4_10B	57	38	0	38	37	0	34	0	23	0	8	1642	0	0	0	0	1	247
V4_11A	26	39	0	13	18	1	49	0	4	0	5	1764	0	0	1	3	5	197
V4_11B	22	46	0	24	21	1	56	0	4	0	9	1765	0	0	2	3	1	171
V4_12A	22	49	0	95	10	0	25	0	8	0	2	1812	0	0	0	0	2	100
V4_12B	21	33	2	195	7	0	21	0	3	0	1	1702	0	0	1	1	5	133

* Samples were normalized to 2125 sequences and are labeled by the following code: Targeted region: V1-V3 and V4, time point: 1-12, technical replicates: A-B

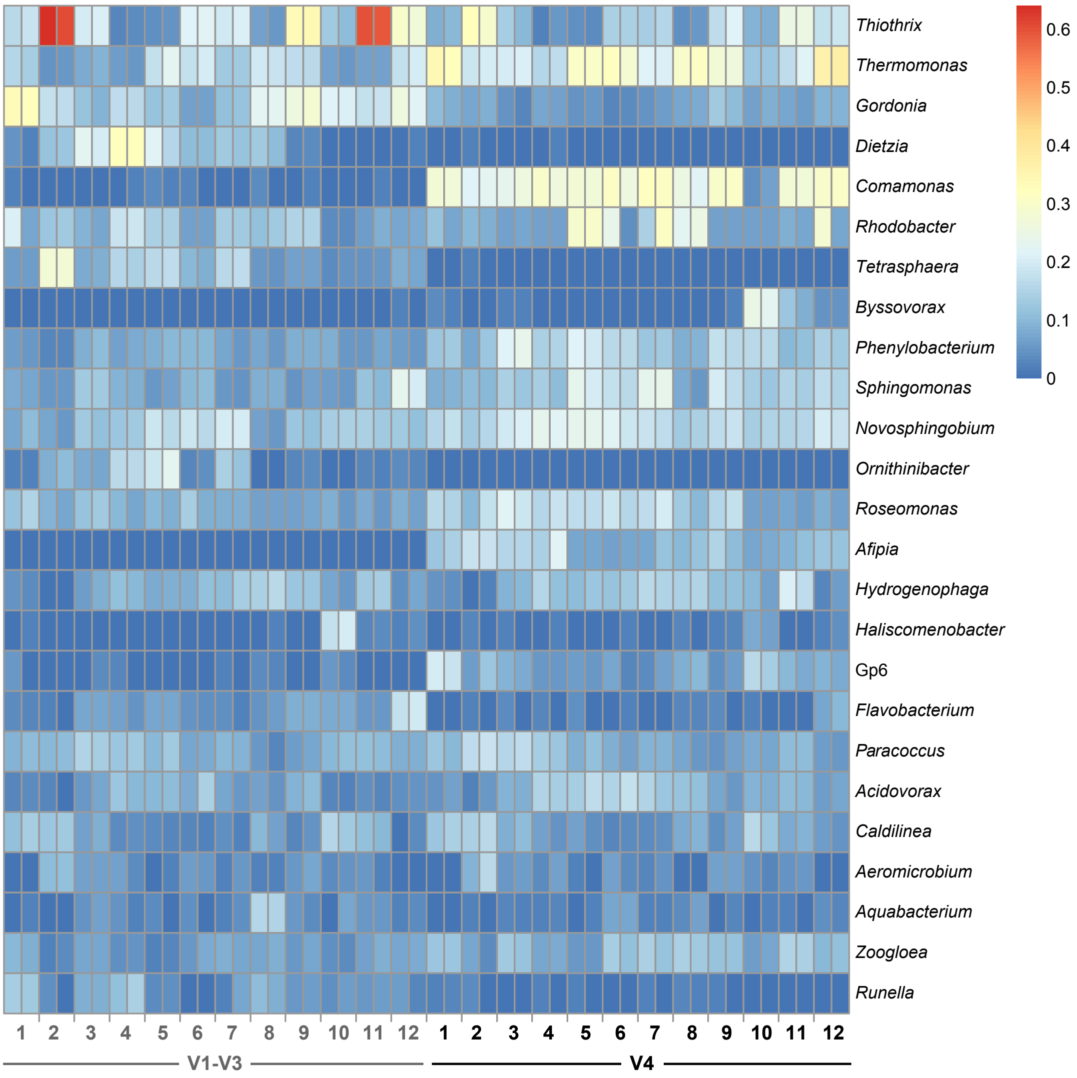

Figure S1. Heatmap of bacterial genera abundance in each time point, based on the analysis of the two 16S rRNA regions. The 25 most abundant genera are shown. Color scale refers to the square root of the abundance of each genus relative to all bacterial sequences.

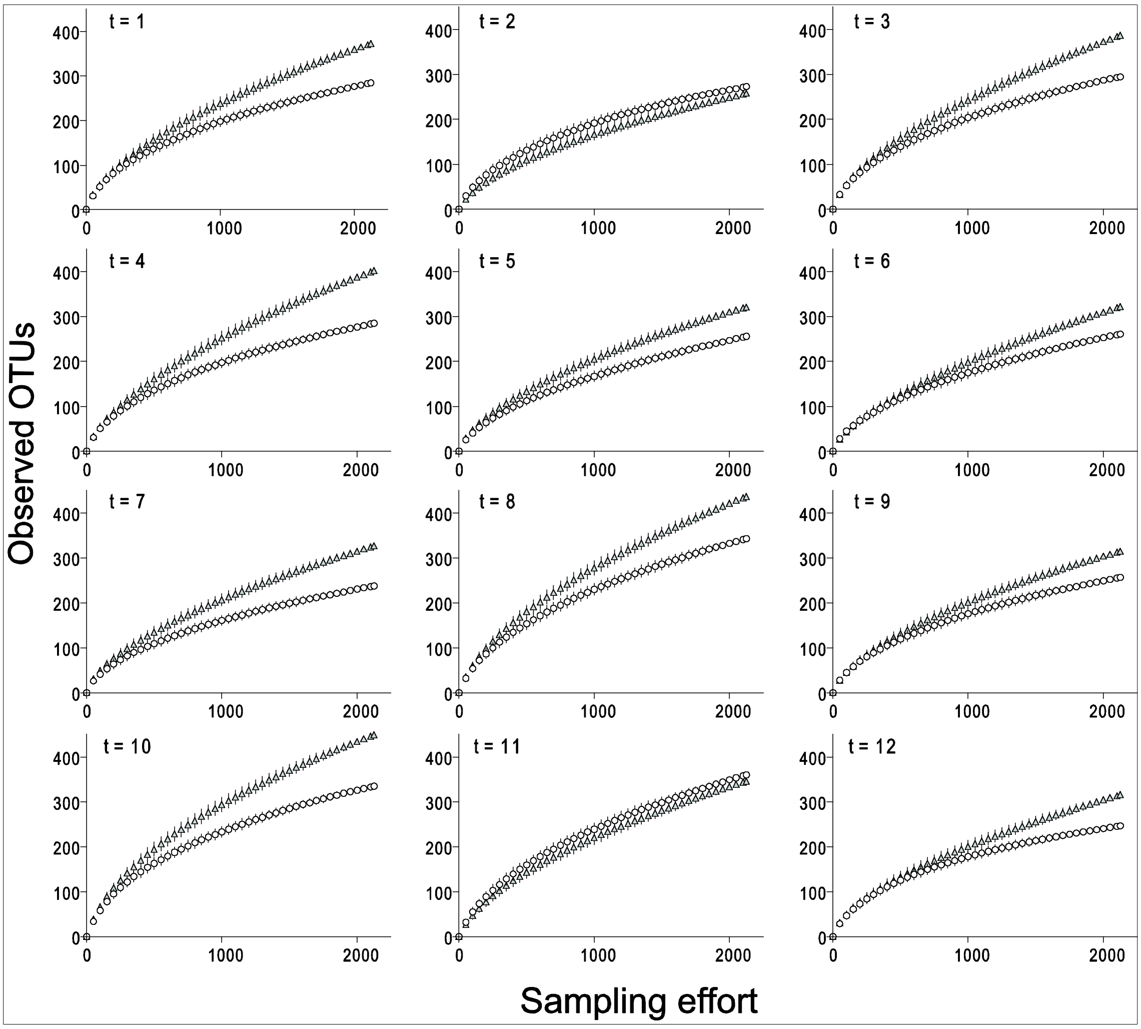

Figure S2. Estimates for bacterial richness are affected by the choice of primers. Rarefaction curves built for OTUs defined at 97% similarity. Each plot corresponds to a time point (month) for V1-V3 (△) and V4 (○). Error bars show the 95% confidence interval derived from the 1000-time iterative sampling.

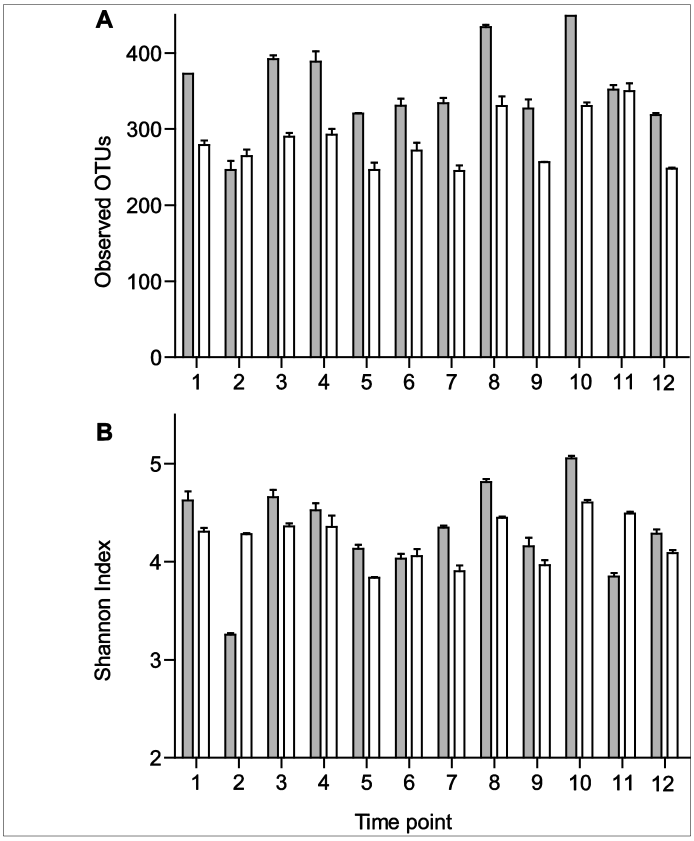

Figure S3. Effect of the different primer sets on the estimation of alpha-diversity. (A) Observed richness and (B) Shannon index were calculated on the normalized samples as average between the two technical replicates. Gray bars represent the values obtained with primers F8-R534 (V1-V3) and white bars, with primers F563-R907 (V4). Error bars show SEM.

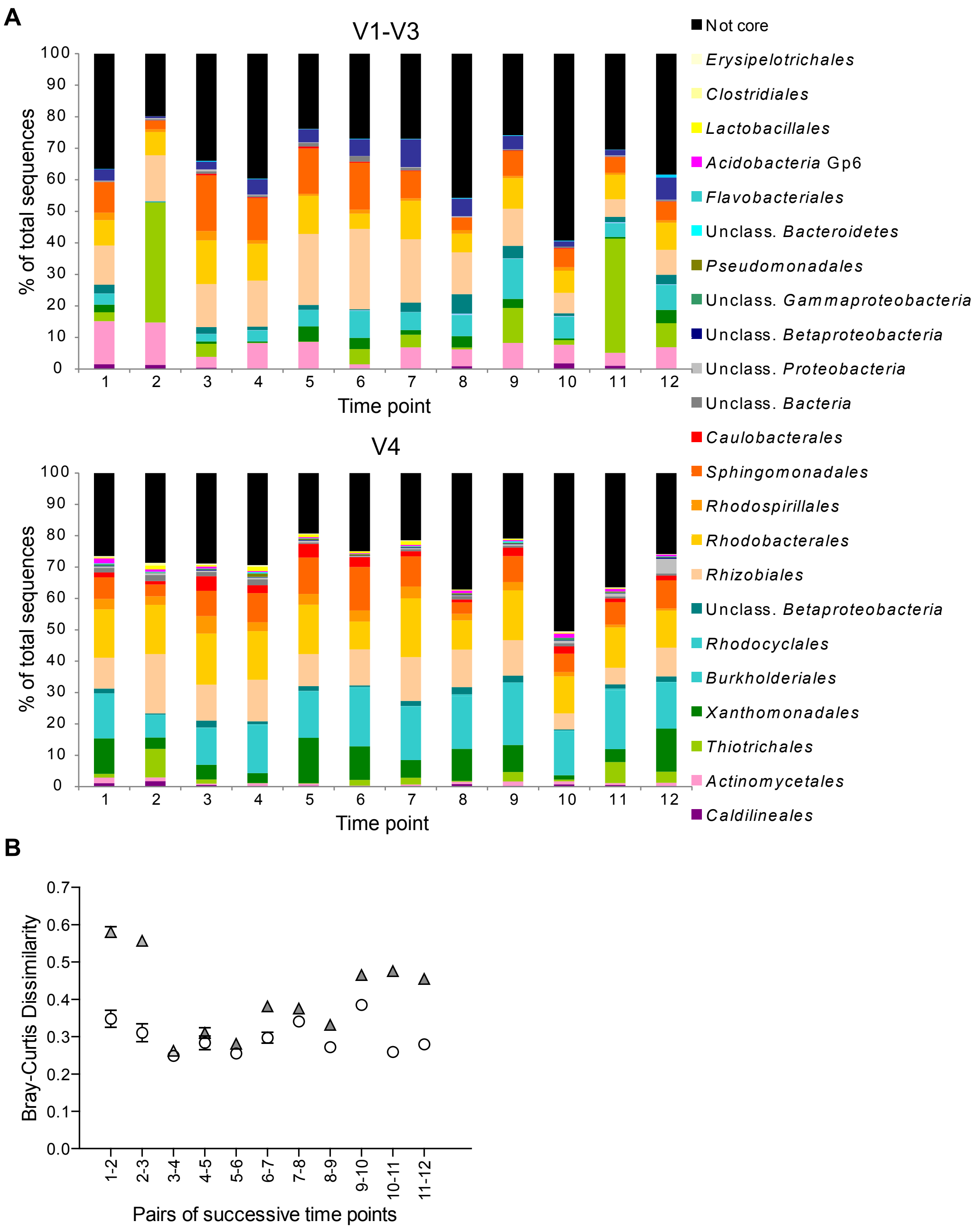

Figure S4. Changes in the relative sequence abundance of bacterial core members. A) Time series of the relative abundances of core members for each 16S rRNA variable region data set. OTUs considered part of the core were detected in all twelve sampling points in at least one 16S rRNA region; all other OTUs were grouped as "not core". Replicates within each 16S rRNA region were averaged for clarity of presentation. B) Moving-window analysis. Symbols indicate the mean of Bray-Curtis dissimilarities between consecutive sampling points within the V1-V3 (△) and the V4 (○) core data sets. Error bars represent standard error.

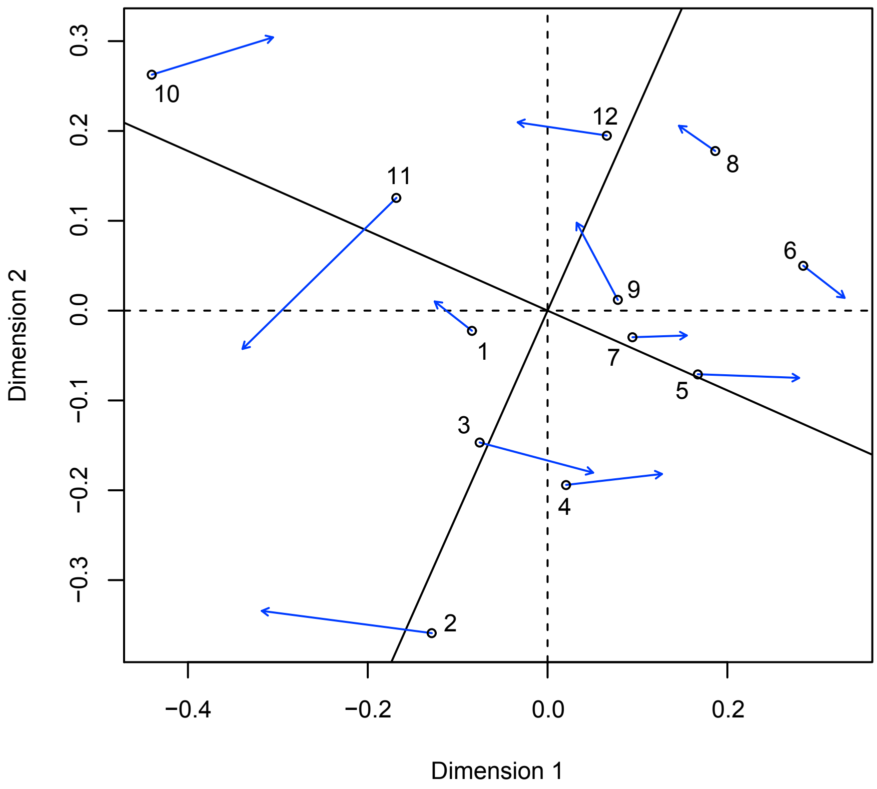

Figure S5. Comparison of beta diversity results based on V1-V3 and V4 16r RNA regions. Procrustes analysis of NMDS ordination plots based on V1-V3 and V4 datasets. Replicates within each 16S rRNA region were averaged for clarity of presentation. Blue lines connect paired samples on the target configuration indicated by circles (V1-V3 region), and the reference configuration (end of arrow, V4 region). Correlation = 0.90, $p < 0.001$.
